# Where have all the flowers gone? A systematic evaluation of factors driving native terrestrial plant decline in North America

**DOI:** 10.1007/s11356-024-34349-9

**Published:** 2024-07-20

**Authors:** Ryan S. Prosser, Richard A. Brain

**Affiliations:** 1 School of Environmental Sciences, University of Guelph, Guelph, ON Canada; 2 Syngenta Crop Protection LLC, Greensboro, NC USA

**Keywords:** Endangered species, Pesticides, Plant diversity, Invasive species, Habitat destruction

## Abstract

Prior to the arrival of Europeans in North America, forest and grasslands individually covered a 3rd of the conterminous United States; however, following the colonial and pioneer periods, respectively, these land cover categories were reduced to 70% and 50% of their original prominence. The dominant driving force for native land conversion was agriculture, which expanded exponentially from the Atlantic to the Pacific, comprising over half the total land area of America at its peak in 1950. However, farmland area has subsequently declined by 25%, so what has been driving native plant declines north of the 30th latitudinal parallel over the past 75 years? Analysis of recovery plans issued by the U.S. Fish and Wildlife Service indicates that of the over 900 plant species “listed” as threatened and endangered the primary driver of decline was invasive species, followed by habitat alteration, and development, which collectively accounted for 93.2% of the primary drivers for listed species. In Canada, these three drivers of decline were the primary drivers for 81% of listed species. Comparatively, herbicides were identified as the primary or secondary driver in 13 out of 1124 cases (1.2%). Given that agricultural land area is contracting in the U.S. and Canada, there appears to be a misconception that agrochemicals are the seminal cause of native plant decline. Here, we explore the individual contribution of drivers relative to the historical events of North America to provide context and perspective as well as focus and prioritize conservation efforts accordingly.

**Supplementary Information:**

The online version contains supplementary material available at 10.1007/s11356-024-34349-9.

## Introduction

With an estimated biodiversity of ~ 500,000 species (Corlett 2016), kingdom Plantae (angiosperms, gymnosperms, ferns, lycophytes, and bryophytes) represents approximately 80% of the biomass on Earth (Bar-On et al. 2018). However, due to a complex interplay between various abiotic and biotic factors, the relative composition of this taxonomic group has changed considerably over geological time (Webb 1987; DiMichele and Phillips 1996; Willis and Niklas 2004). Here, we comparatively evaluate those changes within the geographical and historical context of the United States and Canada. During the Holocene, epoch humanity began to influence the plant communities of North America (Springer et al. 2010; Lyons et al. 2016; Gajewski et al. 2019), which intensified dramatically following the transatlantic arrival of Europeans, whose subsequent activities signaled the genesis of the “Anthropocene” epoch. When the Spanish conquistadors landed in 1492, followed later by the French, British, and Dutch, the process of biological globalization encompassing North America had begun. European ships brought settlers, cultures, technology, diseases, plants, and animals to the new world and returned precious metals, goods, and commodities to the old; this process is popularly referred to as the Columbian exchange (Crosby 2003). These events marked the beginning of the colonial landscape transformation of North America. In addition to changes in the landscape, other factors such as introduced species, disease, climate change, and environmental contaminants have also played a role in the decline of terrestrial plant diversity to varying degrees (Tilman and Lehman 2001; Vellend et al. 2017).

A number of studies have concluded that indigenous communities actively managed vegetation on the landscape in North America for thousands of years before contact with Europeans (Whitehead and Sheehan 1985; Delcourt 1987; Butzer 1992; Krech 1999; Bonnicksen 2000; Abrams and Nowacki 2008; Denevan 2011; Gajewski et al. 2019). For example, fire was used by indigenous groups in North America to promote the growth of plant species that produced seeds, nuts, or berries, to promote the growth of forage for game, to clear underbrush in forests, to prepare areas for cultivation of crops, and to hunt (Bonnicksen 2000; McShea and Healy 2002; Fowler and Konopik 2007; Courtwright 2011). In the eastern U.S., there is evidence that indigenous peoples managed forests to promote the growth of mast trees, as nuts and acorns were an important source of carbohydrates (Abrams and Nowacki 2008). The removal of competing plant species through the use of fire, girdling, and cutting along with the cultivation of beneficial trees species resulted in pre-colonial forest dominated by oak, chestnut, hickory, beech, and/or walnut trees (Delcourt et al. 2004; Abrams and Nowacki 2008).

This early influence of indigenous peoples on plant communities in North America was measurable, but inconsequential relative to the subsequent mass transformation that began with the colonization of North America by Europeans (Butt et al. 2005; Lightfoot et al. 2013). At the time of colonial settlement in 1630, it is estimated that forests covered ~ 1 billion acres (~ 414 million hectares) of land in the U.S., nearly half (44%) of the total (Oswalt et al. 2019). Three centuries later in 1907, forest cover had been reduced by 27% to ~ 760 million acres (~ 307 million hectares) and has remained relatively stable since then (USDA 2001). This land use transition was primarily at the behest of agricultural expansion. From 1700 to 1990, the area of cropland in the U.S. east and west of the Mississippi increased from no large-scale cultivation to ~ 200 million acres (~ 80 million ha) and 321 million acres (130 million ha), respectively (Ramankutty and Foley 1999). Conner et al. (2001) estimate that grasslands once covered roughly half of the contiguous US (~ 1 billion acres; 0.4 billion ha), though between1850 and 1950 this area declined by 260 million acres (105 million ha), primarily via conversion to cropland (Conner et al. 2001). A further 27.2 million acres (11 million ha) of these western grasslands were lost in the subsequent 40 years, though 36% (9.8 million acres; 4 million ha) were attributable to conversions to uses other than cropland (Conner et al. 2001). In Canada, the area of cropland increased to 133 million acres (54 million ha) while forest and prairie each declined by > 74 million acres (> 30 million ha) (Ramankutty and Foley 1999). These changes in the landscape of North America have consequences for plant communities through the conversion of diverse native communities to monocultures.

In the last 30 to 40 years, cropland, which maintains minimal plant biodiversity, has been converted to urban land use (e.g., housing, industrial, commercial, transportation infrastructure), which maintains even less plant biodiversity (Francis et al. 2012). The U.S. lost nearly 2000 acres (> 800 ha) of farmland each day from 2001 to 2016 (Hunter et al. 2022). Canada lost nearly 600,000 acres (239,000 ha) of farmland between 1986 and 2006 and the area of farmland decreased by 3.2% from 2016 to 2021 (Statistics Canada 2008, 2022). In the U.S., “land in farms” peaked at nearly 1.2 billion acres (470 million ha) in 1950 subsequently decreasing to ~ 880 million acres (363 million ha), a drop of ~ 23%. Agriculture is no longer consuming habitat, whereas urban expansion is. This transition is typified by a naturally occurring phenomenon referred to as an environmental Kuznets curve (Stern 2017), which characterizes a shift from agricultural- to industrial-based economies, facilitated by a concurrent intensification in agricultural production (Stern 2017). Stated simply, agricultural expansion is a function of socioeconomic developmental status. Initially developing nations experience environmental degradation at the behest of agricultural expansion; however, an inflection point occurs when agricultural production exceeds demand enabling rural residents to migrate to urban centers and specialize in services and industries. This conversion is followed by a retraction of agricultural land conceded to the interests of urbanization and conservation. Changes in the landscape caused by human activities have played a significant role in plant diversity decline in North America, but habitat loss and degradation are not the only potential driver of plant diversity.

European settlement not only catalyzed large-scale habitat loss and degradation across North America but also facilitated the introduction of non-native flora and fauna, resulting in adverse consequences for native plant species (Kaufman and Kaufman 2023). When the Mayflower set sail from England destined for Plymouth Rock near the tip of Cape Cod, MA, the ship not only contained foreign organisms in the form of Pilgrims but many other taxa as well, including plants. Native species are endemic to a specific geography, originating and developing naturally in their surrounding habitat, whereas non-native (or alien) species are introduced, and include invasive species, “whose introduction causes or is likely to cause economic or environmental harm, or harm to human, animal, or plant health” (Simpson and Eyler 2018). The conterminous United States now contains 6675 non-native taxa, including 3988 species of plants, representing 60% of the total number (Simpson and Eyler 2018). Many agricultural species (e.g., domesticated animals, and plants introduced for crops or horticulture) are non-native including staple crops such as peaches, apples, spinach, carrots, peanuts, oranges, rice, wheat, and soybean. Several of these corps now cover vast swaths of land and have displaced native species as a function of habitat loss at the behest of the agricultural frontier. Unlike crop species, which were intentionally introduced to support a growing nation, invasive species of plants, animals, fungi, bacteria, and viruses have wrought havoc on the remaining habitat not consumed by agriculture, as well as agriculture itself. Estimates suggest that invasive species cost North America $26 billion per year since 2010, up from $2 billion per year in the early 1960s (Crystal-Ornelas et al. 2021). Since 1960, the collective cost associated with invasive species introductions in the U.S. totaled $1.22 trillion, where most costs were associated with invasions of terrestrial habitats ($643.51 billion, 53%) with agriculture being the most impacted sector ($509.55 billion) (Fantle-Lepczyk et al. 2022). The economic cost of invasive species globally has been estimated at $1.288 trillion over the past 50 years (Zenni et al. 2021).

Prominent examples in North America include Dutch elm disease (*Ophiostoma ulmi*), emerald ash borer (*Agrilus planipennis*), brown marmorated stink bug (*Halyomorpha halys*), Japanese beetle (*Popillia japonica*), spotted lanternfly (*Lycorma delicatula*), Asian citrus psyllid (*Diaphorina citri*), feral hogs (*Sus scrofa*), and kudzu (*Pueraria montana*). The Japanese beetle alone is responsible for nearly half a billion dollars in damage to trees, crops, and grasses annually in its larval and adult life stages (USDA 2015). By effectively consuming, displacing, and destroying native species, non-native species of plants, animals, and microbes have caused the extinction of more native species than nearly any other threat (Pimentel et al. 2000).

Climate change may exacerbate the effect of introduced species and disease, along with increasing the frequency of drought, and/or fire, which all can adversely affect plant diversity in North America. There is evidence that climate change can drive the local extinction of plant species (Buse et al. 2015; Wiens 2016; Panetta et al. 2018). Climate change can also influence the risk of plant disease outbreak by altering pathogen evolution and host–pathogen interactions, and expediting the emergence of new pathogenic strains, as well as modifying pathogen range and increasing the spread of disease to new areas (Singh et al. 2023). The potential implications for primary productivity, global food security, environmental sustainability, biodiversity, and the environment are ominous; annual crop yield loss attributed to pathogens and pests alone is estimated at $220 billion USD (Singh et al. 2023).

The discovery of 2,4-dichlorophenoxyacetic acid (2,4-D) by independent research groups in the US and UK in the 1940s (Troyer 2001) helped launch the “Green Revolution” through synthetic chemistry. Herbicides are biologically active compounds specifically designed to manipulate or control undesirable vegetation (i.e., weeds), which compete with desirable vegetation (i.e., crops) for mineral nutrients and water resources. Some herbicides are non-selective or “burn-down,” whereas others are selective between monocots and dicots, though regardless of the mode of action, given that non-target plants are metabolically, biochemically, and physiologically analogous to target plants (weeds), there are clearly potential risks associated with off-field movement of these chemicals (Kudsk and Streibig 2003). Not surprisingly, some research suggests that herbicides can contribute to declines in plant biodiversity (Marshall 2001; Strandberg et al. 2017; Brühl and Zaller 2021) though other studies indicate that plant biodiversity is either unaffected or enhanced when exposed to certain herbicides (Sullivan and Sullivan 2003).

The goal of this study is to examine the different drivers of plant diversity loss in North America outlined above to determine which are the greatest contributors to decline. Prioritization of the drivers of biodiversity loss can ensure that efforts to manage threats to biodiversity are as effective as possible (Carwardine et al. 2012; Mantyka-Pringle et al. 2016). It is important to focus resources on the major drivers and ensure we do not miss the forest for the trees.

## Methods

According to the Canadian Endangered Species Conservation Council’s Wild Species 2020 report, there are 5324 species of vascular plant in Canada (Canadian Endangered Species Conservation Council 2022). The combined number of species of native vascular plants in Canada and the United States (U.S.) is 15,447 (Ulloa Ulloa et al. 2017). In the U.S., 938 species of plant are listed as threatened or endangered; 171 listed as threatened and 767 listed as endangered (USFWS 2023b). In Canada, 206 species of plant are listed as of special concern, threatened, or endangered (Government of Canada 2023). For comparison, 737 and 568 species of animals are listed as of special concern, threatened, or endangered in the U.S. and Canada, respectively (Government of Canada 2023; USFWS 2023b). While this data on plant species at risk of extinction is concerning, listing of a species under legislation in Canada (Species-at-Risk Act) and the U.S. (Endangered Species Act) initiates a process of identifying threats to the listed species and assembling a plan for recovery (Government of Canada 2019; USFWS 2023c).

Data collected by the governments of Canada (Environment and Climate Change Canada and Parks Canada) and the U.S. (U.S. Fish and Wildlife Service) on plant species listed as of special concern, threatened, or endangered in each country was used to identify the most important threats to each listed species. Both countries develop recovery plans for listed plant species and the recovery plans outline the major factors/causes of the species endangerment. For each listed plant species, the identified factors driving the decline of that species were placed in order of importance from primary to quinary. Five drivers of decline were not identified for all listed species. Only the drivers outlined in recovery plans were listed for each species. Twelve different factors were considered based on what was used in the recovery plans from Canada and the U.S. The factors were development, non-native species, fire, drought, extreme weather, climate change, habitat alteration, disease, military activity, harvesting, recreational activities, and pesticides. Activities considered under the factor of development were urban development, recreational (e.g., golf courses), military development, transportation infrastructure, or dam construction. The factor of non-native species included both non-native plant species (e.g., invasive plant species), non-native invertebrate (e.g., plant pest), or non-native vertebrate (e.g., introduced herbivore). Habitat alteration included activities that would fall under agriculture, logging, or mining. Military activity constituted any training that would have a detrimental effect on endangered plant populations, e.g., live fire training. Harvesting included collection for the nursery trade, consumption, illegal trade, and/or hobby collection. Recreational activities that were identified as a potential factor for plant species decline were the use of all-terrain vehicles and hiking.

With respect to uncertainty, recovery plans vary considerably in temporality, both in Canada and the U.S. For example, according to the USFWS, the first plant species listed as endangered under the ESA, which includes the San Clemente Island paintbrush, San Clemente Island larkspur, San Clemente Island broom, and San Clemente Island bush-mallow, were designated in 1977. Consequently, some risk drivers may be understated (e.g., climate change, invasive species) and others overstated (obsolete pesticides that have since been banned or restricted) relative to contemporary conditions. If new information has not been incorporated into older recovery plans, the prioritization of the drivers may change. Additionally, several of the recovery plans do not explicitly rank the drivers of decline for a particular species, which can introduce an element of subjectivity given the ranking may vary based on interpretation. Therefore, the reliability of the underlying source data varies between listed plant species. Deference was made to the governmental institutions that generated the source data regardless of issuance date.

## Results and discussion

### Ranking of factors that drive decline of plant species

**Fig. 1:**
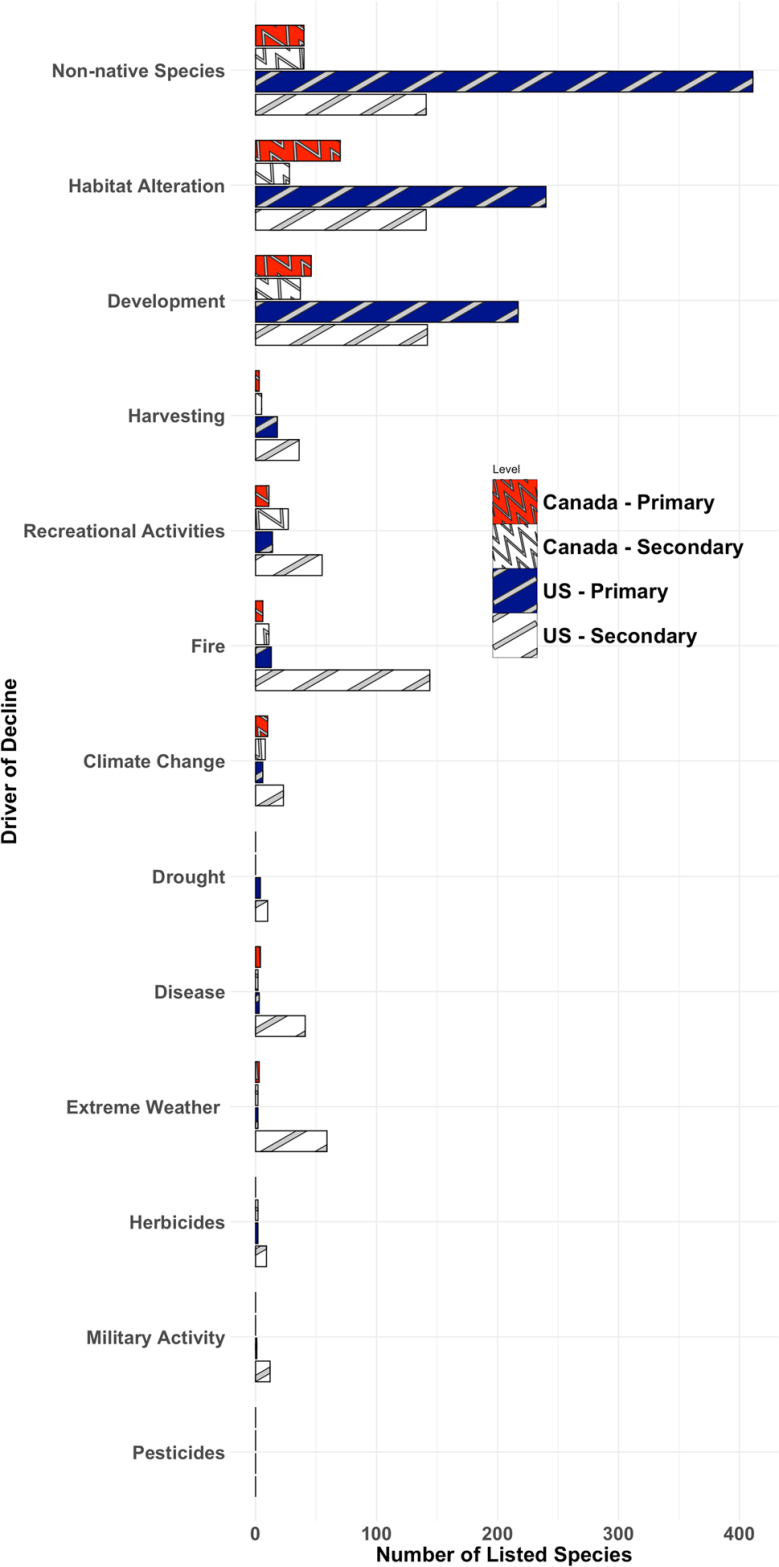
The number of plant species listed under the Species-at-Risk Act by the Government of Canada which each driver has been identified as the primary (red wave) or secondary (white wave) cause of decline. The number of plant species listed under the Endangered Species Act by the United States Fish and Wildlife Service for which each driver has been identified as the primary (blue stripe) or secondary (white strip) cause of decline

In the U.S., the primary driver of decline for threatened or endangered terrestrial plant species was non-native species, followed by habitat alteration and development (Fig. 1). Non-native species were the primary driver for nearly the same number of plant species (407) as for habitat alteration (240) and development (217) combined (Fig. 1 and Table S1). Interestingly, fire was the secondary driver for a greater number of listed species (144) in the United States compared to habitat alteration (141), development (142), and non-native species (141) individually (Fig. 1 and Table S1). Habitat alteration was the primary driver of decline for the greatest number of endangered, threatened, or species concern terrestrial plant species (70) in Canada followed by development (46) and non-native species (40) (Fig. 1 and Table S2).

An explanation for non-native species being the primary driver of decline for the greatest number of plant species in the United States is the relatively large number of listed species on islands in the Pacific Ocean, e.g., Hawaiian Islands, Guam. Nearly half of the listed species (458 species) in the United States are located in the U.S. Fish and Wildlife Service’s Pacific region (Region 1), which includes American Samoa, Guam, Hawaiian Islands, Idaho, Northern Mariana Islands, Oregon, and Washington (Table S3). The majority of listed species in Region 1 are located on the Hawaiian Islands and Guam. Island ecosystems are particularly sensitive to the introduction of invasive non-native plants and animals (Donlan et al. 2003; Reaser et al. 2007; Russell and Kaiser-Bunbury 2019; Elton 2020). Island plant species are at risk of extirpation from the introduction of non-native species due to native species relatively limited distribution, small population size, and the absence of coevolution with herbivores and competitors (Vermeij 1991; Paulay 1994; D’antonio and Dudley 1995). The combination of Guam and the Hawaiian Islands containing a relatively large number of endemic plant species and the invasion of these islands by invasive non-native plant, invertebrate, and vertebrate species has resulted in a relatively large number of terrestrial plants on these islands being classified as threatened or endangered (Smith 1985; Cowie 2000; Nogueira-Filho et al. 2009; Rogers et al. 2017). For example, European explorers released European pigs (*Sus scrofa scrofa*) and goats (*Capra hircus*) on the Hawaiian Islands to supply future voyageurs with a source a protein. Pigs and goats were followed by the introduction of cattle (*Bos taurus*), sheep (*Ovis aries*), and axis deer (*Axis axis*) (Lohr and Lepczyk 2010). The foraging of these herbivorous mammals has had a profoundly negative impact on terrestrial plant species endemic to the Hawaiian Islands (Spatz and Mueller-Dombois 1972; Aplet et al. 1991; Nogueira-Filho et al. 2009; Weller et al. 2018).

Herbicides or insecticides were identified as the primary or secondary driver of decline for a total of 12 listed plant species in the U.S. and 2 species in Canada (Fig. 1 and Table S1 and S2). Herbicides, insecticides, or pesticides were identified as a driver of any importance (primary to quinary) for a total of 46 listed plant species in the U.S. and 10 species in Canada (Table S1 and S2). *Marshallia mohrii* (Mohr’s Barbara’s buttons) and *Physaria filiformis* (Missouri bladderpod) are the two listed plant species for which herbicides were identified as a primary driver of decline (Table 1). Herbicides are a primary driver of decline in *M. mohrii* and *P. filiformis* because many identified populations are located along roadways and other types of rights-of-way where the spraying of herbicides is used to control weed plant species which could result in exposure to these two listed species (USFWS 1988, 2022a). Herbicides were identified as secondary drivers to listed plant species, again, due to inadvertent exposure from their use in rights-of-ways to control vegetation or inadvertent exposure from spray drift when used in agriculture (USFWS 1990, 1994c, b, a, 1995, 1996, 1998, 1999, 2005) (Table 1). Herbicides were not identified as a primary driver of decline for any plant species-at-risk in Canada, but herbicides were identified as a secondary driver for two plant species (Table S2). The inadvertent exposure to herbicides used for the removal of invasive plant species may be a driver of decline for *Psilocarphus brevissimus* and *Phlox speciosa* ssp*. occidentalis* (ECCC 2013, 2017). Insecticides were identified as a potential driver of decline for 4 listed plant species in the U.S., but not identified for any species in Canada (Table S1 and S2). *Styrax platanifolius* ssp. *texanus* is an obligately xenogamous species, which means this listed plant species requires pollination by honey bee (*Apis mellifera*), American bumble bee (*Bombus pensylvanicus*), or California carpenter bee (*Xylocopa californica*) (USFWS 2019). Consequently, insecticides have been identified as a potential driver of decline for this plant because they can adversely affect the pollinators that this plant species requires for reproduction (USFWS 2019). For the same reasons, insecticides have been identified as a driver of decline for the other three listed plant species in the U.S., i.e., *Eremalche kernensis*, *Argythamnia blodgettii*, and *Caulanthus californicus* (Table S1) (USFWS 1998).

**Table 1:** Terrestrial plant species listed as threatened or endangered by the United States Fish and Wildlife Service or the Government of Canada with herbicides or pesticides identified as primary or secondary drivers of decline

Scientific name	Common name	Primary driver	Secondary driver
*United States*			
*Marshallia mohrii*	Mohr’s Barbara’s buttons	Herbicides	Development
*Physaria filiformis*	Missouri bladderpod	Herbicides	Non-native species
*Boltonia decurrens*	Decurrent false aster	Habitat alteration	Herbicides
*Cereus eriophorus* var. *fragrans*	Fragrant prickly apple	Habitat alteration	Herbicides
*Clematis morefieldii*	Morefield’s leather flower	Development	Herbicides
*Conradina glabra*	Apalachicola rosemary	Habitat alteration	Herbicides
*Lesquerella lyrata*	Lyrate bladderpod	Non-native species	Herbicides
*Neostapfia colusana*	Colusa grass	Habitat alteration	Herbicides
*Opuntia treleasei*	Bakersfield cactus	Development	Herbicides
*Sisyrinchium dichotomum*	White irisette	Development	Herbicides
*Styrax platanifolius* ssp. *texanus*	Texas snowbells	Non-native species	Insecticides
*Thalictrum cooleyi*	Cooley’s meadowrue	Development	Herbicides
*Canada*			
*Psilocarphus brevissimus*	Dwarf Woolly heads	Habitat alteration	Herbicides
*Phlox speciosa* ssp*. Occidentalis*	Showy Phlox	Development	Herbicides

When searching through recovery plans for each listed species in the United States and Canada, drivers could not be found for 7 and 13 species, respectively. The species for which drivers could not be found are listed in the Supplementary Information (SI) (Table S4 and S5).

### Non-native species

#### Non-native plants

Non-native or alien species can be broken out into two primary groups, innocuous and invasive. Introduced species in the U.S. and Canada account for 16.5 and 26% of the total number of species, respectively (Marvin et al. 2009) and these introductions can be either unintentional (e.g., ship ballast) or intentional (e.g., agronomically valuable or aesthetically pleasing species). Of the 13,575 plant species identified in the continental U.S., 9402 are native, 2397 are endemic, 1201 are alien, and 755 are invasive according to an analysis of the U.S. Department of Agriculture (USDA) PLANTS database (Bradley et al. 2015).

##### Innocuous non-native plants

Innocuous plants generally do not outcompete or put native species biodiversity at risk. However, vast swaths of native forests and grasslands have been converted to cropland and pasture for agricultural production. “Land in farms” increased from virtually nothing at the advent of colonial settlement in North America to a peak of 1,161,419,720 acres (470 million ha) in 1950, subsequently decreasing by ~ 23%; there are 1,893,801,241.60 acres (~ 766 million ha) in the contiguous U.S. (Brain et al. 2023). “Cropland” similarly increased from a very modest land area devoted to subsistence agriculture by native tribes to a peak of 478,315,094 acres (194 million ha), before declining ~ 17% (Brain et al. 2023). Introduced crops have thus covered as much as a quarter of the landscape of the contiguous U.S.

Henry Yonge, the Surveyor-General of Georgia, first planted soybean (*Glycine max*), native to East Asia, on his farm in 1765 at the request of the seaman Samuel Bowen, formerly of the East India Company, who brought seeds to Savannah Georgia, from China via London (Hymowitz and Harlan 1983). In 2023, soybeans covered over 82,770,018 acres (33 million hectares), or nearly 5% of the contiguous U.S. (note soybeans tend to overlap, with the “native” crop species, corn). Other introduced crop species such as sugarcane, rice, barley, sorghum, and wheat covered 755,698, 2,861,741, 2,951,505, 6,618,999, and 53,281,370 acres (0.3, 1.2, 2.7, and 22 million hectares) in 2023, respectively, according to the USDA (2024a). The impact of non-native crop species is broadly considered under the auspices of habitat loss due to agricultural expansion, thus, here we will focus primarily on invasive species.

##### Invasive non-native plants

Invasive plants are considered one of the greatest threats to cropland, rangeland, aquatic areas, and wildlands in the U.S. (Mullin et al. 2000). Estimates suggest that more than 40% of all imperiled native U.S. plants and animals are at risk of extinction due to invasive species introductions (McGrath 2005). Invasive plants have contributed to the decline of 42% of threatened and endangered species and considered the main cause of decline for 18% (USFS 2024). In 2006, it was estimated that invasive plants covered over 100 million acres (40 million ha) of land in the U.S., expanding at a rate of 3 million acres (1.2 million ha) each year (National Invasive Species Council 2008). However, more contemporary estimates from the U.S. Forest Service peg the number of acres containing invasive plants at 133 million acres (54 million ha) (as big as California and New York combined), in federal, state, and private ownerships (USFS 2024). Moreover, the same source indicates that 3.6 million acres (1.5 million ha) (equivalent to the size of Connecticut) of national forests are being choked out by invasive species, and that these species are advancing at a rate of 1.7 million acres (0.7 million ha) per year (two-thirds bigger than the size of Delaware) across the nation in all directions. Next to mammals, plants were found to be the second costliest invaders of the U.S. at $190.45 billion, primarily affecting agriculture (Fantle-Lepczyk et al. 2022). As discussed in Beaury et al. (2023), the primary source of invasive plants into the U.S. is the horticultural industry, where hundreds of plant species categorized as invasive are still available for sale through mail order and retail nurseries. Beaury et al. (2023) analyzed a data set of 89 invasive plants sold by 672 nurseries and found that horticulture could be seeding invasions for 73 species studied and facilitating climate-driven range expansion of 25 species. Moreover, 55% of the invasive plant species were sold within 21 km of an observed invasion (Beaury et al. 2023). Climate change of + 2 °C is predicted to increase the range of invasive plant species by an average of 213 km (Evans et al. 2024).

Famed wildlife ecologist Aldo Leopold described cheatgrass (*Bromus tectorum*) as a scourge of the Western USA (Leopold 1949). This invasive annual is a prolific seed producer (150,000 seeds/lb) (Klemmedson and Smith 1964) and was first introduced to North America from Europe in the mid- to late-1800s as a contaminant in seed and straw (https://www.usgs.gov/centers/forest-and-rangeland-ecosystem-science-center/science/cheatgrass-and-medusahead). The common name for this species derives from its germination timing, specifically that it can take root while native perennial grasses are still dormant during the winter, thus “cheating.” Cheatgrass displaces native species, which cannot compete, and kills biological soil crusts (lichens, mosses, and cyanobacteria) resulting in soil erosion, resource competition, altered nutrient cycling, and modified local fire cycles due to premature die-off relative to other native grass species (Freeman et al. 2014). Cheatgrass is expanding by hundreds of acres per day (USFWS 2017) and now covers over 100 million acres (40 million ha) (https://www.nps.gov/arch/learn/nature/cheatgrass.htm); estimates suggest that this invasive plant will expand its occupied range by 5 × in the Great Basin between the Wasatch and Sierra Nevada mountains within 30 years. The resultant changes in food availability and microhabitat have had a dramatic impact on wildlife (e.g., the greater sage grouse) (Freeman et al. 2014).

Similarly, stinknet (*Oncosiphon pilulifer*) is considered one of the most invasive, non-native plants to become established in the southwestern United States (Hedrick and McDonald 2020). This winter annual, which can grow in dense mats (up to 6 seedings per square cm), originated from South Africa and was first discovered in southern California in 1981, then Arizona in 1997 (Hedrick and McDonald 2020). Stinknet is also a prolific seed producer (250 seeds per flower head) and germinates quickly, outcompeting native winter annuals and perennial vegetation resulting in changes to wildlife habitat, and increased fire risk (Hedrick and McDonald 2020). Moreover, the seeds are very small and light facilitating dispersal by wind, vehicles, water, equipment, people, and wildlife (Hedrick and McDonald 2020). Stinknet patches can cover several to hundreds of acres and this invasive species continues to spread with large populations now occurring in most Southern California counties within 20 years of its discovery, extending from the coast to the desert (McDonald 2019). Stinknet also threatens the fragile Sonoran biome in New Mexico as Maricopa County is heavily infested, and the invader has also been observed in Northern Mexico and Las Vegas (Duncan and Shaw 2023).

If cheatgrass is the scourge of the West, then kudzu (*Pueraria montana*) is its counterpart in the Southeast. Originating in China, Japan, India, and Micronesia, this perennial leguminous twining vine was first introduced at the 1876 Centennial Exposition in Philadelphia, PA (Miller and Edwards 1983; Forseth and Innis 2004). Thought to be effective for mitigating soil erosion among other uses, 85 million seedlings were provided to landowners by government agencies prior to 1950, though twenty years later kudzu was declared a weed (Forseth and Innis 2004). So popular was kudzu that clubs were formed in the 1940s and festivals held to crown “kudzu queens” (Miller and Edwards 1983). Now kudzu covers over 7 million acres (3 million ha) of land (Blaustein 2001) and is expanding at a rate of 124,000 acres (50,000 ha) per year (Mitich 2000). Many key traits of *P. montana* contribute to its ability to spread rapidly and dominate natural communities, literally carpeting the landscape (Forseth and Innis 2004). Kudzu is considered a structural parasite, clinging to other plants for support to reach high light levels at the top of the forest canopy, and its growth form enables stem elongation rates between 3 and 19 cm per day, totaling 20 to 30 m per growing season (Forseth and Innis 2004). Economic impacts of kudzu on forests are estimated at between $100 and 500 million per year (Blaustein 2001).

Purple loosestrife (*Lythrum salicaria*) is also considered one of the most common invasive species in the U.S., infesting more than 400,000 acres of federal land, including wetlands, marshes, pastures, and riparian meadows (Zimdahl and Brown 2018). This attractive invasive plant kills sedges, cattails, and bulrush, thus crowding out native plants that provide food and cover for waterfowl (OMNRF 2022). First introduced from Eurasia in the early nineteenth century via contaminated solid cargo ship ballast and later imported deliberately as an ornamental plant, this species can now be found in every state in the contiguous U.S. except for Florida. *L. salicaria* is highly aggressive due to its rapid growth and hardiness, prolific reproduction (both sexual and vegetative), and lack of natural pests and competitors (Reinartz et al. 1987). Like other invasives, this perennial is a prolific seed producer generating 300,000 seeds per stalk from each of 15 to 20 stalks per plant ranging in height from 2 to 2.5 m in length (Reinartz et al. 1987). A single hectare of land can contain as many as 200,000 stalks and produce ~ 60 billion seeds per year (Reinartz et al. 1987). Consequently, this deceptively beautiful ornamental can form dense stands of monoculture that can outcompete native species that reduces local biodiversity, puts rare species at risk, and provides little to no value to wildlife.

Another example includes the introduced plant species garlic mustard (*Alliaria petiolata*) which has contributed to the decline of American ginseng (*Panax quinquefolius*), a long-lived herbaceous perennial species listed as endangered in Canada (Wixted and McGraw 2009, 2010).

#### Non-native invertebrates

Non-native invertebrates can directly impact native terrestrial plants through herbivory (Fajvan and Wood 1996; Lynch 2004; Poland and McCullough 2006; Ancheta and Heard 2011) and/or indirectly by transmitting disease, adversely affecting native pollinators, or altering plant community structure (McClure 1989; Boettner et al. 2000; Brasier 2000; Colla et al. 2006; Eschtruth et al. 2006; Morin et al. 2007; Kenis et al. 2009).

An example of non-native invertebrate herbivory on threatened native plant species is *Rhinocyllus conicus*. *R. conicus* is a species of true weevil native to Eurasia and North Africa that has been introduced to North America to be used as a biocontrol agent of noxious thistle species (Louda et al. 1997, 2005; Rose et al. 2005). However, the non-native weevil has caused the decline of rare native thistle species in North America such as *Cirsium canescens* (Platte thistle) and *Cirsium pitcheri* (Pitcher’s thistle) (Louda et al. 1997, 2005). Several slug and snail species have been introduced to the Hawaiian Islands through the horticulture industry (e.g., *Achatina fulica*, *Deroceras laeve*, *Bradybaena similaris*, *Veronicella cubensis*) (Cowie 2000, 2001; Cowie et al. 2008). Joe and Daehler (2008) found that non-native slug species (*D. laeve*, *Limacus flavus*, *Limax maximus*, *Meghimatium striatum*) contribute to the decline in populations of the endangered native plant species *Cyanea superba* and *Schidea obovata* due to herbivory. These slug species can also be a significant barrier to restoration of rare plants on the Hawaiian Islands (Joe and Daehler 2008).

Along with causing plant diversity decline through direct herbivory, non-native invertebrates can indirectly adversely impact native plant species. Several non-native insect species have been identified as the vector of several devastating plant diseases. The small European elm bark beetle (*Scolytus multistriatus*) has played an important role in the rapid spread of the fungal pathogen, *Ophiostoma novo-ulmi*, responsible for Dutch elm disease (Brasier 2000; Jacobi et al. 2013; Santini and Faccoli 2015). *Scolytus multistraitus* lay eggs underneath the bark of elm trees and the larvae feed in the phloem of the tree (Santini and Faccoli 2015). When mature *S. multistraitus* emerge from bark, the conidia of *O. novo-ulmi* on their body are transported to other elm trees. Another example is the European beech scale (*Cryptococcus fagisuga*), which facilitates the infection of beech trees by the fungal pathogen *Neonectria faginata* (Morin et al. 2007). Infection by *N. faginata* produces severe cankers on the trunk of the tree and deformity of stems which will lead to death of trees.

Non-native pollinators can adversely affect native plant communities by either more effectively pollinating invasive plant species and/or being less effective pollinators of native species (Morales et al. 2017; Debnam et al. 2021). For example, the invasive plant *Solanum torvum* (turkey berry) exhibited pollen limitation when pollinated by native pollinators but not when pollinated by non-native pollinators, e.g., *Euglossa viridissima* (orchid bee), in Florida (Liu and Pemberton 2009). In this example, non-native pollinators indirectly impact native plant communities by promoting pollination of *S. torvum*, which can outcompete native plant species resulting in changes to native plant communities (Liu and Pemberton 2009). Page and Williams (2023) observed that the introduction of non-native honey bees caused lower seed production in the wildflower *Camassia quamash* found in montane meadows in the U.S. Non-native bees species reduced the availability of nectar and pollen for native bee species that are more effective pollinators for *C. quamash* (Page and Williams 2023).

#### Non-native vertebrates

As mentioned previously, the introduction of vertebrates, specifically ungulates, to the Hawaiian Islands has had considerable consequences for native terrestrial ecosystems (Stone and Loope 1987; Weller et al. 2011, 2018). These introduced ungulates (e.g., pigs, cattle, deer) have become important drivers of decline for native plant species on the Hawaiian Islands through herbivory and physical disturbance due to their movement on the landscape (Thaxton et al. 2010; Cole and Litton 2014).

Introduced ungulates have also been a driver of decline in terrestrial plant diversity in the continental U.S. and in Canada. The majority of threatened or endangered terrestrial plant species in the Southwestern U.S. have been directly or indirectly adversely impacted by the introduction of cattle (USFWS 1985, 1994d, 2018, 2020; Jones 2000). Domestic cattle can directly adversely impact native plant species by grazing on the plants or by trampling plants (USFWS 1985, 1987, 2020; Kimball and Schiffman 2003). However, the presence of domestic cattle can also indirectly be a driver of decline for native plant species by disturbing topsoil, reducing the establishment of seedlings, increasing the potential of erosion and flooding, decreasing the quantity and diversity of pollinators, and/or changing natural fire regimes (Jones 2000; Scheintaub et al. 2009; Lazaro et al. 2016; USFWS 2018, 2020; Blanchette 2019). Overgrazing by cattle can reduce the fuel load in grassland ecosystems, leading to a reduction in the frequency of fires (Sala and Maestre 2014; Wilcox et al. 2018). Maintenance of the natural fire regime is critical to grassland ecosystems in North America through preventing the invasion of woody plant species and non-native grasses into grasslands and stimulating the growth of native prairie grasses and forbs (USFWS 2018; Wilcox et al. 2022). The disruption of natural fire regimes has been identified as a threat for several threatened or endangered plant species in the U.S., e.g., *Hoffmannseggia tenella*, *Ambrosia cheiranthifolia*, *Festuca ligulata*, *Streptanthus bracteatus*, and *Pectis imberbis* (USFWS 2018, 2022b, 2023a, d). It is important to recognize the overgrazing from cattle and other domestic ungulates is not the only factor disrupting natural fire regimes. Anthropogenic fire suppression and climate change also alter the natural cycle of relatively frequent low-intensity fires that are required by grassland ecosystems and the listed plant species in these ecosystems (Keeley et al. 1999; Paysen et al. 2000; Archer and Predick 2008; Hurteau et al. 2014).

Non-native vertebrates that have an adverse effect on terrestrial plant diversity in North America are not limited to ungulates. The introduction of the brown tree snake (*Boiga irregularis*) to the island of Guam has indirectly had an extraordinary impact on plant diversity (Mortensen et al. 2008). The brown tree snake is responsible for the extirpation of the 12 native forest bird species on the island of Guam (Savidge 1987; Rodda et al. 1992; Wiles et al. 2003; Rogers et al. 2017). Approximately 70% of the tree species rely on birds to consume their fruits which is critical to seed dispersal for these tree species (Mortensen et al. 2008). The loss of the dispersal services of frugivorous birds has contributed to the decline of several threatened and endangered plant species on Guam, e.g., *Tabernaemontana rotensis*, *Claoxylon marianum*, *Eugenia bryanii*, and *Maesa walkerii* (Guam Plant Extinction Prevention Program 2024).

Non-native species can also interact to cause adverse effects on native plant species. Feral pigs in the Hawaiian Islands are an interesting case study of how non-native species can interact to amplify their effects on native species. As mentioned above, feral pigs contribute to the loss of native plant biodiversity through foraging and landscape alterations. Feral pigs were present on numerous Pacific islands before the arrival of Europeans but the population densities were relatively low due to the low abundance of animal protein available to pigs (Barret and Stone 1983). However, the introduction of earthworms and dung beetles to the islands provided an abundant source of protein to pigs, which lead to the range expansion of feral pig populations (Griffin 1978; Doing 1982; Anderson 1994). Feral pigs also aid in the expansion of non-native plant species by carrying seeds on their coats (e.g., *Carex alligata*, *Paspalum conjugatum*) and in their guts (e.g., *Psidium cattleyanum*) (Doing 1982). Feral pigs can also disperse seeds from the non-indigenous and nitrogen-fixing *Myrica faya* tree in forests (Aplet et al. 1991). The increased nitrogen in soil due to *M. faya* can cause an increase in earthworm abundance, and consequently increase the abundance of protein available to feral pig populations (Aplet 1990). The introduction of these non-native species has compounded the loss of native plant diversity on the Hawaiian Islands.

### Habitat alteration and development

**Fig. 2:**
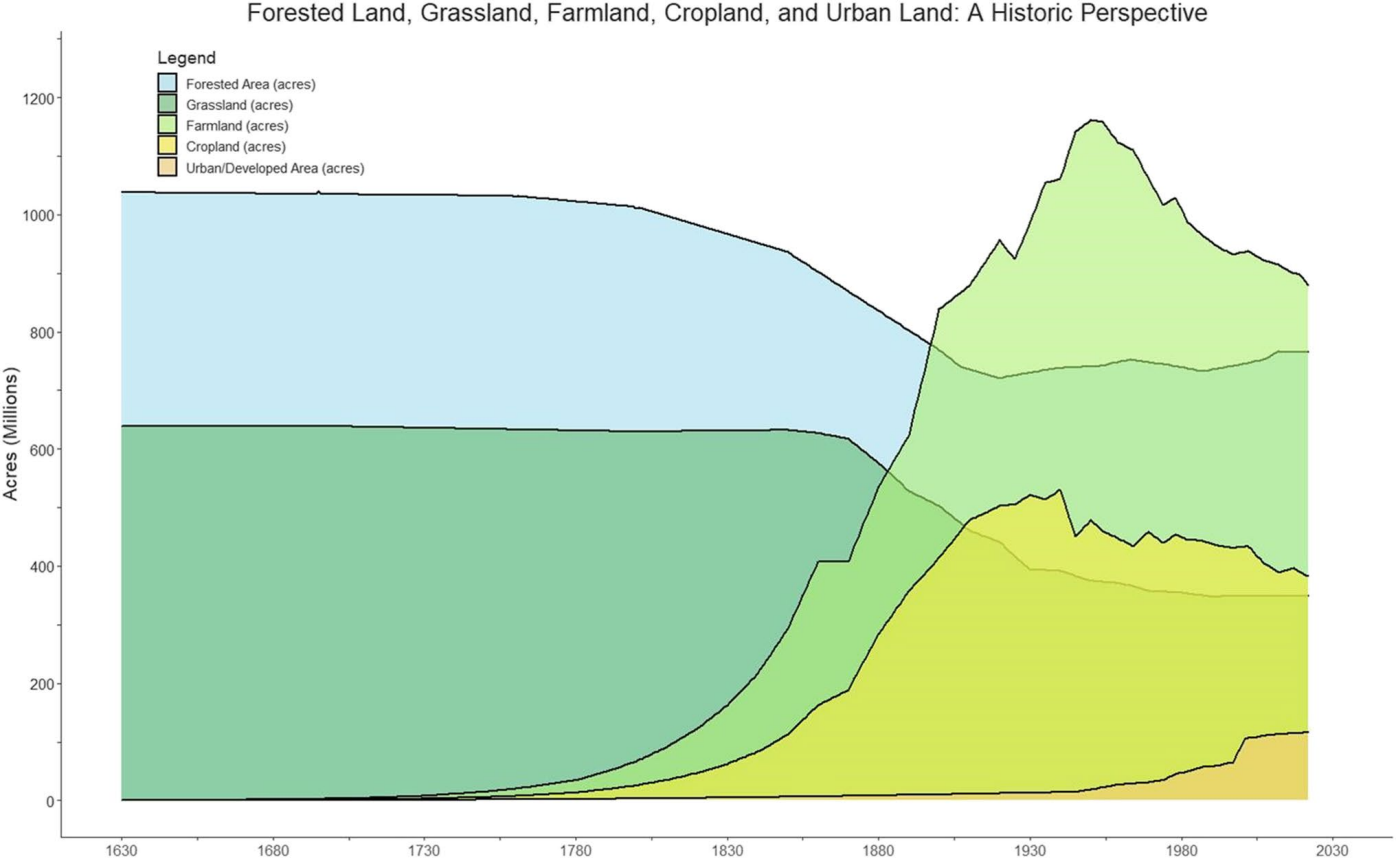
Historical trends in land use area (acres) of forest, grassland, farmland, cropland, and urban cover since 1630 within the United States. Farmland (“land in farms”) and cropland area were sourced from the United States Department of Agriculture Census of Agriculture records (USDA 2024b). Farmland and cropland acreage estimates prior to 1850 were extrapolated based on population relative to land area identified in the first Census of Agriculture. Forest area was sourced from the United States Forest Service USFS (2014) and Alvarez (2007). The grassland data layer (historical estimates of grassland cover in the continental US) was reconstructed from Wang et al. (2023) and Conner et al. (2001). These two datasets were derived from methodologies using a combination of cover of the conterminous United States (CONUS) satellite imagery and qualitative analysis of trends and driving forces of forest and grassland changes (e.g., population, per capita income, US Homestead act, tax law, agricultural production factors). Grassland datasets were interpolated to a yearly interval for graphical purposes. Urban land was derived from two data sources, due to availability of historical data: (1) Economic Research Service, 2007 (1945–1997) and (2) all “developed” land classes in the National Land Cover Dataset (2001–2021)

During the Little Ice Age (1450 to 1850 AD) prehistoric small-scale agricultural societies (i.e., subsistence farming) in North America caused widespread ecological change, including decreased forest cover and increased sedimentation due to settlement reorientation and intensification of corn production long before the advent of European settlement (Stinchcomb et al. 2011). Therefore, the landscape of North America was neither pristine nor undisturbed prior to the onset of the Columbian exchange (Crosby 2003). However, the advent of colonial establishment in, and expansion over, North America, originating from the Atlantic, signaled the genesis of wholesale landscape transformation that reverberated across the continent, concluding at the Pacific. As the composition of the landscape changed, so too did the ethnic mosaic. Waves of colonial settlers diffusing across the conterminous United States in pursuit of “manifest destiny” not only catalyzed the erosion of soil, environment, and native flora and fauna, but also endemic culture, tradition, lifestyle, and language. The progressive subjugation of native Americans and dispossession of their lands is emblematic of the coincidental augmentation of the natural landscape. Technically the “American Indian Wars” (1609–1890) began the moment British colonizers first set foot on the shores of the new world at present-day Jamestown, Virginia in the late 1500s, albeit following the arrival of Jacques Cartier at Gaspé Bay in 1534 along the coast of Quebec. Here, we delineate two transformative periods, the early colonial period (1600 to 1800 AD) typified by the mass felling of trees in the eastern U.S. and Canada, and the pioneer period (1800 to 2000 AD) typified by mass cultivation of native grasslands across the prairies. Both periods were primarily resultant from the establishment and expansion of agriculture (Fig. 2). The singularity of broad-scale agricultural expansion in North America can be primarily traced back to the Jamestown colony, where the merchant John Rolfe introduced a new variety of tobacco in 1612 AD (Brain and Anderson 2020).

Subsequent to the establishment of the Virginia and Plymouth Companies, forests were harvested *en masse* for housing, export to Europe, ship building, and provisioning for agriculture (Brain and Anderson 2020). The USDA estimates that forest cover represented nearly half the total land area of the U.S. in 1630 (~ 1023 million acres or 414 million ha; ~ 46%) but dropped precipitously by a quarter of a billion acres (100 million ha) over the next three centuries to ~ 34% (~ 754 million acres) in 1910 (USDA 2024b) (Fig. 2). The amount of forest and woodland east of the Mississippi decreased from 484 million acres (196 million ha) to 227 million acres (92 million ha), a decrease of nearly 250 million acres (100 million ha) (Ramankutty and Foley 1999). Grassland west of the Mississippi decreased from ~ 640 million acres (258 million ha) to ~ 350 million acres (141 million ha), a decrease of > 270 million acres (> 110 million ha) (Ramankutty and Foley 1999). Nearly two-thirds (~ 565 million acres; 229 million ha) of this geography was located east of the Rocky Mountains, pre-settlement, with over 90% now under private ownership. The proportional decline in natural habitat in certain regions of North America is much greater than at the continental scale. In Southern Ontario, 80% of the land was forested in the middle of the eighteenth century before European settlement, but the forested area has declined to ~ 15% at present (Butt et al. 2005). However, in accordance with the environmental Kuznets curve (Mills and Waite 2009; Franklin and Ruth 2012; Leblois et al. 2017; Sarkodie and Strezov 2018; Brain and Anderson 2020), forest area has been relatively stable over the past century, despite a population increase of over threefold.

During the Revolutionary War, the first treaty between the United States government and Native Americans was signed at Fort Pitt, Pennsylvania, in 1778. This treaty recognized sovereignty of the Delaware Nation and territorial rights; however, the accord was quickly broken by the relentless advance of European-American settlers into what is now Ohio, as well as conflict with the British. This common theme of broken promises, amplified by the Indian Removal Act of 1830, continued until the closing of the Western frontier around 1890. Once the American Indian had been fully subjugated by the United States Army, the country was completely opened to settlement. Farrell et al. (2021) estimate that native Americans have been dispossessed of 99% of their original lands. Significant historical events that contributed to the transformation of the Great Plains include the Louisiana purchase, the Expedition of Lewis and Clark, smallpox, the Homestead Act, buffalo hunters, the railroad, and the plow. Briefly, the United States purchased 530 million acres (215 million ha) of territory (nearly doubling the size of the country) from France (Napoleon) in 1803 for $15 million, which encompassed nearly the entirety of the Great Plains, and the expedition of Lewis and Clark (commissioned by President Thomas Jefferson) in 1804 charted the geography across the newly purchased territory all the way to the Pacific. Smallpox, as well as other diseases including measles, typhus, and scarlet fever, collectively killed an estimated 50 to 90% native Americans (Roberts 1989). The Homestead Act of 1862 catalyzed settlement west of the Mississippi by granting 160 acres (65 ha) to adult heads of families for virtually nothing. Bison, which once numbered in the 10’s of millions across the great plains, were driven to near extinction by buffalo hunters sanctioned by the U.S. Army. The transcontinental railroad, built between 1863 and 1869, spanning 1911 miles (3075 km) across the country rapidly increased settlement, and the steel plow, introduced by John Deere in 1837, made possible the turning of soil in areas thought to be unsuitable for farming. Consequently, the native American population virtually disappeared from the central plains, and the grassland prairies were converted to pasture and farmland; these events realized the pioneer period. Between 1850 and 1950, grassland area declined by over a quarter of a billion acres (100 million ha) west of the Mississippi River (Conner et al. 2001) (Fig. 2). Smith (1992) estimates that at least 97% of the original 240 million acres (97 million ha) of tallgrass prairie have been lost. In Iowa alone, which is the nation’s number one row-crop producer (corn and soybeans), less than 0.1% of the original 30 million acres (12 million ha) of prairie remain (Smith 1992). A detailed timeline of approximate settlement dates for regions of the tallgrass prairie is provided by Smith (1992), spanning from 1790 to 1920. The plow is described by Smith (1992) as symbolic of the demise of the prairie. Following further technological advancements in farm machinery (e.g., plows, harrows, planters, cultivators, reapers, and threshers) from the 1840s to 1870s, the unconditional conversion of native grassland to cropland was inevitable by the turn of the century. Disturbing the native soil also made possible the establishment and spread of weed and invasive plant species (e.g., blue-grass and dandelions) throughout the Great Plains (Smith 1992).

The natural landscape in Canada has also been subject to these two transformative periods. The grassland ecosystems in the Canadian Prairie provinces and in the dry valleys of southern interior British Columbia have been considerably altered from the western expansion of European settlers. The vast majority of the original grassland is gone, making these grassland ecosystems in Canada one of the most altered in North America (AAFC 2010). The majority of grasslands and their associated wetlands have been converted to agriculture. Over the last 150 years, approximately 65% of the land within the Parkland Prairies, Central Grassland, and Eastern Prairies ecoprovinces in Canada has been converted to agriculture and this is without considering grasslands that have been converted to natural pasture and rangeland (Fig. S1) (Statistics Canada 2024). A large percentage of the Carolinian forests of Central Canada and southwestern Ontario and the mixed Acadian forests in the Maritime provinces have also been converted to agriculture. For example, 58% and 39% of the Huron-Erie plans and Great Lakes-St. Lawrence Lowlands ecoprovince has been converted to agriculture, respectively (Fig. S1).

In pursuit of opportunity and a better life, manifest destiny filled the prairies with American settlers motivated by moral virtue, ideology, and faith. The dominant occupation at the dawn of the nineteenth century was farming, where employment in agriculture accounted for nearly three quarters of the U.S. work force (Lebergott 1966). By 1850, the urban population began to expand, and the labor dominance of agriculture dropped to 55%, then to 40% by 1900, and further to just over 10% by 1950 (Lebergott 1966). Between 1950 and 1990, the number of self-employed and family farmworkers declined by 74% (7.60 to 2.01 million) according to the USDA’s National Agricultural Statistical Service (NASS). As of 2023, the U.S. Bureau of Labor and Statistics pegs direct on-farm employment at ~ 2.2 million jobs in the U.S. or ~ 1 percent of the total labor market. At the peak of agricultural expansion, “land in farms” covered 1,161,419,720 acres (~ 470 million ha) of the U.S., fully half of the available 2.3 billion acres (930 million ha) of American land (Fig. 2). However, “land in farms” has been steadily decreasing ever since and currently stands at 880,100,848 acres (356 million ha), a drop of nearly 25% (Fig. 2). The 2022 Census of Agriculture indicates that the U.S. lost ~ 20 million acres (8 million ha) of farmland between 2017 and 2022 (USDA 2024a). So where is all this land going if not being farmed? The American Farmland Trust estimates that between 2001 and 2016, 11 million acres (4.5 million ha) of farmland and ranchland was converted to urban and highly developed land use (4.1 million acres) or low-density residential land use (∼7 million acres) (Freedgood et al. 2020). Urban land area has been steadily increasing since the middle of the nineteenth century and currently occupies over 100 million acres (40 million ha) based on the National Land Cover Dataset (NLCD), or ~ 70 million acres (28 million ha) according to USDA ERS. This number may seem somewhat trivial relative to the total land area of the U.S.; however, the precipitous decline in farmland has only been offset by production gains as total factor productivity has increased nearly threefold since 1948 (Brain et al. 2023). At present, for every one individual employed (“on-farm”) in agriculture (2,179,000 farmers/hired farm workers), there are 150 dependent census-enumerated souls (334,914,895 of them) to support, and most of these dependents reside in urban spaces (80% of the general population). Urban spaces typically expand at the acquiescence of the most fertile agricultural land given the prerequisite dependency of urban development on agricultural production (Rees 1992; Imhoff et al. 2004; Satterthwaite et al. 2010; Tauger 2010). “The vertical city of the nineteenth century, compact and intensive in its land-use, has been surrounded by a horizontal, land-devouring suburbia” (Manners 1965). Over a third of the land lost to urbanization is prime farmland (soil capability class I and II) (Plaut 1980). In Canada, between 2000 and 2015, the amount of urban land use rose by 11% and this urban expansion was mainly at the expense of prime agriculture land located in the Huron-Erie Plains, Georgia Depression, and Great Lakes-St. Lawrence Lowlands ecoprovinces (Fig. S1) (Statistics Canada 2024). In 2001, almost half of the urban land in Canada was on what was dependable agricultural land (Statistics Canada 2005). Between 1971 and 2001, the amount of urban land use increased by 96% in the Canada (Statistics Canada 2024). Bigelow and Borchers (2017) estimate that urban land use grew at more than twice the rate of population between 1945 and 2012 in the U.S. Moreover, 9% of urban land area and 39% of all houses in the conterminous U.S. are located within the urban-wildlands interface (Radeloff et al. 2005). Not surprisingly, the most attractive high-amenity developments are typically located within spaces exhibiting rich biodiversity given that access to outdoor recreation and natural landscapes are main drivers for human consumers (Brain and Anderson 2020). Unintended ecological consequences of extensive development include habitat fragmentation, direct threats to wildlife, and biodiversity declines (Brown et al. 2005; Radeloff et al. 2005). Expansion of urban land use over agricultural is estimated to be equivalent to the caloric requirement of 16.5 million people or ~ 6% of the US population due to the loss of net primary productivity (Imhoff et al. 2004). Commensurate with the sequence depicted by the environmental Kuznets curve, America’s agricultural footprint expanded rapidly and relentlessly westward at the expense of native forests and grasslands. However, once the nation crested the apex of the agrarian frontier, farmland predominance ceded territory back to nature, as well as to the impervious creep of concrete and asphalt.

### Disease

Just as smallpox impacted the native American (human) fauna of the U.S., so too has the disease been a factor in the decline of native terrestrial flora in North America, albeit to a much lesser extent. Non-native fungal pathogens have been particularly destructive. A previously mentioned example is Dutch elm disease, a particularly destructive disease caused by the introduction of the ascomycete fungi *Ophiostoma* sp. to North America that has resulted in a drastic decline in populations of native elms trees (Karnosky 1979; Campana and Stipes 1981). A fungal pathogen is also the most important threat to the endangered coniferous tree *Pinus albicaulis* (whitebark pine) in North America (Vogler and Charlet 2004). *Cronartium ribicola* was introduced from Europe and causes white pine blister rust in *P. albicaulis* (Rochefort 2008). The critically endangered species *Torreya taxifolia*, considered the rarest conifer in North America, is at risk of extinction due to the fungal pathogen *Fusarium torreyae* and potentially other *Fusarium* sp., drastically reducing the size of remaining populations in the southeastern U.S. (Aoki et al. 2013).

Disease can also indirectly affect terrestrial plant diversity. Two fungal pathogens (*Ceratocystis lukuohia*, *Ceratocystis huliohia*) have caused the death of *Metrosideros polymorpha* (‘ōhi ‘a lehua tree), one of the most common native tree species on the Hawaiian Islands, across large areas of the islands (Fortini et al. 2019). More than half of the range of 147 (62.8%) of the 234 endangered native plant species in the Hawaiian Islands have conditions that are suitable for infestation by *C. lukuohia* (Fortini et al. 2019). This leads to concern that the mass mortality events of the ‘ōhi ‘a lehua tree caused by *C. lukuohia* could have negative consequences on these threatened or endangered native plant species due to changes in the ‘ōhi ‘a forest ecosystems (Fortini et al. 2019). Non-native ungulates (e.g., feral pigs) have also been found to accelerate the spread of *C. lukuohia*, which provides another example of compounding factors that can affect native plant diversity (Fortini et al. 2019; Perroy et al. 2021).

### Extreme weather events and climate change

The “Anthropocene” epoch (albeit not officially recognized or agreed upon in terms of geological timing) popularly denotes the current geological age, emblematic of the period during which the dominant influence on climate and the environment has been human activity. Whether you are a believer or skeptic of climate change, extreme weather events have been increasing (https://science.nasa.gov/climate-change/extreme-weather/). The main reason that extreme weather events can be an important factor in the loss of native terrestrial plants is due to the limit distribution of many rare species. Extreme winds and/or precipitation can quickly destroy isolated populations from directly damaging vegetation or from causing flooding, landslides, and/or storm surges. In 2018, the Atlanta Botanical Garden planted 700 trees of the critically endangered *Torreya taxifolia* only to lose all of the seedlings as a result of Hurricane Michael toppling hardwood trees (Emerson 2019). Extreme heat and drought are also an important threat to terrestrial plant diversity, particularly in the southwestern U.S. (Bernardo et al. 2016; McIntosh et al. 2020).

The incidence of extreme weather events posing a risk to terrestrial plant diversity cannot be discussed without considering climate change. Climate change will result in an increase in extreme weather events (Stott 2016). For example, Sheffield and Wood (2008) projected that drought frequency in the southeastern U.S. will increase two to threefold due to climate change. The change in temperature and precipitation regimes will vary across regions in North America, but regardless of region, it will result in changes in the distribution of critical habitat for threatened and endangered plant species (Wrobleski et al. 2023). For example, reduction in the frequency of freezing temperature, length of the frost-free season, and increased minimum temperatures will change the geographic and elevation boundaries of the Mojave, Sonoran, and Chihuahuan deserts which will be accompanied by changes in the ranges of plant species (Archer and Predick 2008). This relatively rapid change in conditions may have dire consequences for threatened and endangered plant species with a limited distribution. Changes in temperature and precipitation patterns due to climate change may also enhance the invasion of ecosystems in the southwestern U.S. by non-native plant species, which will also negatively impact native plant species (Archer and Predick 2008; Abatzoglou and Kolden 2011). Increases in the frequency and duration of drought may also increase herbivory on native plant species from non-native vertebrates. For example, the endangered cactus *Echinocactus horizonthalonius* var. *nicholii* (Nichol’s Turk’s head cactus) found only in the Sonoran Desert has declined in certain areas due to increased herbivory by sheep (McIntosh et al. 2020). Increased drought conditions in the Sonoran Desert due to climate change is projected to increase herbivory, which would further impact this endangered cactus (Shryock et al. 2014; McIntosh et al. 2020).

Climate change may also cause changes in the distribution of invasive invertebrate, vertebrate, and plant species across North America (Simberloff 2000; Mainka and Howard 2010). For example, Colautti and Barrett (2013) observed that the invasive plant *L. salicaria* (purple loosestrife) was able to rapidly adapt to changes in climate leading to range expansion. Climate change is also predicted to expand the northern range of *Lymantria dispar dispar* (European gypsy moth or spongy moth), which is classified as one of the most destructive invasive species in the Northern hemisphere (Gray 2004; Hennigar et al. 2007; Logan et al. 2007; McManus and Csóka 2007; Tobin et al. 2007; Régnière et al. 2009). The range of invasive plant pathogens, including white pine blister rust, sudden oak death disease, pitch canker (*Fusarium circinatum*), and Port-Orford-cedar root disease, may also expand due to a warming climate (Poland et al. 2021).

### Pesticides

The field of synthetic chemistry exploded following the second world war. Among pesticides, the organochlorines, which technically includes the herbicide 2,4-dichlorophenoxyacetic acid (2,4-D), and more notoriously, the insecticides dichlorodiphenyltrichloroethane (DDT), dieldrin, endrin, chlordane, heptachlor, etc., dominated the market from 1945 to the early 1970s. DDT is generally considered the first synthetic pesticide, its insecticidal mode of action discovered by Swiss chemist Paul Hermann Müller in 1939. However, as explained by Troyer (2001), “The discovery of the first synthetic hormone herbicides, 2,4-D, 2,4,5-T, and MCPA, initiated the agricultural revolution and modern weed science.” Following discovery of the phenoxy acetic acids, other consequential herbicides were developed and registered including atrazine (1958), paraquat (1964), glyphosate (1974), metolachlor (1976), and mesotrione (2001). These compounds are designed/selected specifically to manipulate biological processes unique to plants, thus presenting potential direct risks to this taxon when applied. In contrast, insecticides affect biological processes unique to animals, thus presenting potential indirect risks to plants via impacts on pollinators, etc. Here, we will focus primarily on herbicides (i.e., direct effects).

After hostilities ended in 1945, insecticides dominated the market; DDT and toxaphene alone comprised a combined 46% of the total amount of pesticides applied in 1964. However, by the end of the decade, herbicide use had outpaced that of insecticides, fungicides, and all other pesticides combined (Fernandez-Cornejo et al. 2014). In an analysis of pesticide use (amount applied) on 21 select crops, Fernandez-Cornejo et al. (2014) found that insecticides accounted for nearly 60% in 1960, but only 6% in 2008, whereas herbicide use grew from less than 20% to over 75% in the same timeframe. With respect to acres of corn, wheat, and cotton treated, herbicides were applied to only ~ 5–10% in 1952 compared to ~ 90–99% in 1980 (Fernandez-Cornejo et al. 2014). Moreover, the same analysis reported that pesticide use rose rapidly from 89 million kg (196 million lbs) of active ingredient (a.i.) applied in 1960 to a peak of 287 million kg (632 million lbs) in 1981. This was largely due to increased crop acreage (primarily corn, wheat, and soybeans) treated with herbicides to control weeds and price declines relative to other pest control practices (i.e., substituting costs for labor, fuel, and machinery use in mechanical weed control) (Fernandez-Cornejo et al. 2014). As of 2008, pesticide use has trended downward to 234 million kg (516 million lbs), where recent fluctuations have been driven primarily by planted acreage (particularly corn, cotton, soybeans, potatoes, and wheat), crop and input prices, weather, pesticide regulations, and the introduction of new technology including genetically modified seed (Fernandez-Cornejo et al. 2014).

Unlike successive generational classes of insecticides, which demonstrate improved toxicity to non-target birds, mammals, and invertebrates, reduced application rates, and more favorable environmental fate profiles, the toxicity of herbicides to non-target terrestrial plants (NTTPs), relative to corresponding applications rates, and inherent soil persistence have remained relatively consistent since the 1940s (Table 2). Although some classes of herbicides such as the sulfonylureas are considerably more toxic to NTTPs than other groups, these compounds are also applied at markedly lower rates agronomically. Thus, in general, the intrinsic risks posed by herbicides to NTTPs have not changed substantially in over 80 years, though some compounds including 2,4,5-T and daminozide (Alar) have been removed from commerce, albeit not related specifically to NTTP concerns.

**Table 2:** Terrestrial plant toxicity, chemical property data, and application rates (agricultural uses) for classes of herbicides in chronological order of introduction. Data were accessed primarily from USEPA ecological risk assessments, where the source documents can be accessed by entering the corresponding docket number in https://www.regulations.gov/

Class/compound		Vegetative Vigor ER25 (lb a.i./A)		Vegetative Vigor NOER (lb a.i./A)		Seedling Emergence ER25 (lb a.i./A)		Seedling Emergence NOER (lb a.i./A)		Log K_ow_		Aerobic soil half-life (d)		Anaerobic soil half-life (d)		Max single application rate for agriculture (lb a.i./A)		Source(s): USEPA Docket Number (EPA-HQ-OPP-), other regulatory documents, and additional peer-reviewed sources
1940s to 1960s																		
2,4-Dichlorophenoxyacetic acid	0.0021		0.00167		0.00081		0.00047		0.1–2.14		1.4–12.4		NA		0.07–4		EPA-HQ-OPP-2012–0330-0047	
Atrazine	0.054		0.0016		0.0098		0.025		2.68		61		NA		0.4–4		Smith et al. (2021) EPA-HQ-OPP-2013–0266-0315	
Trifluralin	0.841		0.50		0.0399		0.0137		4.83–5.34		116–381		25–59		0.63–4		EPA-HQ-OPP-2012–0417-0021	
Paraquat	0.0208		0.0106		0.635		0.171		< − 2.35		Stable (> 180)		Stable (> 60)		0.5–1.5		EPA-HQ-OPP-2011–0855-0128	
Diuron	0.002		0.001		0.08		0.047		2.68		372		1000		0.2–2.4		EPA-HQ-OPP-2003–0349-0002	
EPTC	0.97		4.07		0.28		0.174		3.34		46.33–51.1		Stable		2.0–14.8		EPA-HQ-OPP-2012–0720-0015	
1960s to 1980s																		
Dicamba	0.000513		0.000261		0.0357		NA		2.21		18		NA		0.125–7.7		EPA-HQ-OPP-2005–0479-0008 EPA-HQ-OPP-2020–0492-0002	
Glyphosate	0.074		0.049		> 5		> 5		< − 3		1.8–109		199–208		0.38–8		EPA-HQ-OPP-2009–0361-0077	
Metolachlor	0.016		0.003		0.0048		0.0010		3.05		14.6–231		45.5		1.26–3.17		EPA-HQ-OPP-2014–0772-0028 USEPA (2007)	
Acifluorfen	0.0038		0.0009		0.032		0.015		1.55		100–200		NA		0.25–0.375		EPA-HQ-OPP-2010–0135-0014	
Trifluralin	0.796		0.25		0.09		0.06		4.83–5.34		116–201		25–59		0.75–4		EPA-HQ-OPP-2012–0417-0003	
Metribuzin	0.002		0.0001		0.01		0.002		1.65		310		NA		0.17–3		EPA-HQ-OPP-2012–0487-0018	
Triclopyr	0.0054		NA		0.062		NA		4.01		6–115		69–170		0.375–9		EPA-HQ-OPP-2014–0576-0026	
1980s to present																		
Glufosinate	0.063		0.05		0.15		< 0.15		< 0.1		8.5–23		37		0.3–1.5		EPA-HQ-OPP-2008–0190-0023	
Mesotrione	0.00023		0.0001		0.0019		0.0005		< − 1		4.3–82.2		4.1–14^a^		0.25		EPA-HQ-OPP-2013–0779-0022	
Chlorsulfuron	0.00001		0.0000008		0.000022		0.0000026		− 0.95		12.1–17.3		NA		0.0078–0.14		EPA-HQ-OPP-2012–0878-0003	
Cloransulam-methyl	0.00004		0.00006		0.00062		0.00050		− 0.365		13		19–59.8		0.032–0.055		EPA-HQ-OPP-2010–0855-0015	
Pinoxaden	0.005		0.002		0.016		0.008		3.2		0.21		NA		0.063		EPA-HQ-OPP-2015–0603-0020	
Clodinafop-propargyl	0.0048		0.0028		0.017		0.00788		> 3		< 1–2.4		NA		0.0632		EPA-HQ-OPP-2012–0424-0020	
Sethoxydim	0.029		0.038		0.078		0.0587		1.65		< 1		> 60		0.28–0.47		EPA-HQ-OPP-2005–0323-0018	

*NA* no risk assessment value available

Some herbicides are broad spectrum, whereas others are selective between monocots or dicots. According to the revised 2020 Herbicide Resistance Action Committee (HRAC) classification system, there are 16 known unique modes of herbicidal action which are broken out by “Group,” broadly reflecting 9 target processes, lipid synthesis inhibitors, amino acid synthesis inhibitors, growth regulators, photosynthesis inhibitors, nitrogen metabolism inhibitors, pigment inhibitors, cell membrane disruptors, seedling root growth inhibitors, and seedling root growth inhibitors (HRAC 2024). These targets are conserved across plant species, though absorption, translocation, metabolism, and receptor binding affinity may differ among species. Consequently, the exposure of NTTPs to any class of herbicide poses potential risks.

There are two primary routes of pesticidal exposure for NTTPs, runoff from treated fields and spray drift from direct application (USEPA 2004). The EPA provides guidance regarding the requisite testing protocols, exposure scenarios, methodologies, and benchmarks to be used for the purposes of risk assessment (USEPA 2004, 2023a; Code of Federal Regulation 2024). Briefly, seedling emergence and vegetative vigor endpoints for NTTPs are derived from greenhouse studies that expose standard crop species via direct overhead application using a track sprayer to either bare soil (seeds below the surface) or seedlings at the 2 to 4 leaf stage, to reflect runoff or drift exposure, respectively. Treated plants are then typically grown for 21 or 28 days and evaluated for morphological and visual effects. The most sensitive endpoint is then typically used for risk assessment. Models that simulate runoff (Pesticide Water Calculator [PWC]; previously Pesticide Root Zone Model—Exposure Analysis Modeling System [PRZM-EXAMS]) and drift (AgDRIFT) are then used to estimate exposure for these scenarios. The Agency calculates the 90th centile runoff concentration from PWC based on estimates of 30 annual peak values, which on a daily basis could actually be as high as the 99.97th percentile (Brain et al. 2015) and relies on conservative deposition fractions from AgDRIFT with generic default assumptions (i.e., high-boom, very fine to fine droplet size distribution), and 90th centile wind speed for ground applications at Tiers I and II (Brain et al. 2019). However, the endpoint derivation is paradoxical relative to how exposure is calculated for both runoff and drift scenarios. Virtually by definition, NTTPs do not experience exposure in the form of direct overhead application given that they are not located within the field boundary (otherwise they would be considered “weeds”). By virtue of being off-field, NTTPs are exposed to runoff and drift as a function of precipitation intensity, soil type, slope, and porosity, as well as windspeed, and direction, and not as a direct function of the field rate delivered under the spray boom, commensurate with the exposure delivered by a track sprayer per the guideline studies (Brain et al. 2015, 2017). Stated simply, the EPA screening-level scenarios do not reflect reality. Correspondingly, most research evaluating herbicidal effects on NTTP biodiversity (Marshall 2001; Sullivan and Sullivan 2003; Strandberg et al. 2017; Brühl and Zaller 2021) has been conducted on plots directly (and successively) sprayed by herbicides, rather than areas adjacent to the field margin, where, by definition, “non-target” terrestrial plants may be present.

While vapor drift can move certain herbicides with relatively high vapor pressures (e.g., dicamba) several kilometers under inversion conditions (Bish et al. 2021), non-target plant effects from physical drift for many common herbicides are typically limited to ≤ 30 ft (~ 9 m) off the field margin as demonstrated by several field drift bioassays (Marrs and Frost 1997; Brain et al. 2017; Moore et al. 2022; Perkins et al. 2022). These studies directly address the paradoxical aspect of the existing risk paradigm by exposing NTTPs downwind to physical drift primarily as a function of windspeed and direction. Potted plants are placed downwind of a field-rate spray application delivered under worst-case conditions (e.g., windspeeds exceeding labelled thresholds, bare soil, multiple spray swaths) and then transferred back to a greenhouse, grown out, and assessed for effects in accordance with standard guidelines. Essentially, this higher-tier design is a combination of the Agency’s Drift Reduction Technology (USEPA 2016) and vegetative vigor protocols (USEPA 2012b). Relative to maximal buffers of 1000 ft (~ 300 m) or more predicted by the Agency’s current framework for many herbicides, real-world empirical data indicates a buffer ≤ 33 × smaller or less from the field margin is sufficiently protective of sensitive NTTPs in most cases. To the authors’ knowledge, there are currently no analogous field runoff bioassay designs evaluating the effects of herbicide runoff simulated under worst-case conditions, in combination with standard seedling emergence protocols (USEPA 2012a), though this may be a valuable area of research to pursue. Boutin et al. (2014) also assessed transects of plants in woodlots adjacent to agricultural fields that had received herbicide applications previously with and without a 15 m (~ 50 ft) buffer and found that “most affected plants were located in quadrats within the 1 to 4 m range, though some effects were noticeable up to 32 m.” Moreover, plants can often recover from herbicide injury (Brain and Hoberg 2016). Consequently, potential effects induced by inadvertent exposure to most synthetic herbicides are not widespread nor permanent, although this is often what is portrayed in the popular media.

Standard guideline protocols for NTTP testing primarily assess vegetative and observational endpoints rather than reproductive endpoints. However, some studies have assessed effects of herbicide exposure on plant reproduction in both fields (Olszyk et al. 2017) and greenhouse-based experiments (Boutin et al. 2014; Mathiassen et al. 2021). Olszyk et al. (2017) assessed the effects of glyphosate and dicamba on directly oversprayed plots containing 9 perennial species from grassland habitats native to the Willamette Valley (Oregon) and found that, generally, 10 to 20% of the field rate was required to affect reproduction (e.g., number of reproductive structures, mature and immature seed production). Boutin et al. (2014) similarly describe a series of greenhouse experiments with several compilations of plant species exposed to fractional rates of glufosinate, chlorimuron-ethyl, and fluroxypyr examining effects on flowering times and number of flowers, deriving a factor of 3 in terms of greater sensitivity of reproductive endpoints relative to vegetative endpoints (biomass). In slight contrast, Mathiassen et al. (2021) exposed 9 non-target terrestrial plant species belonging to 6 different families to 4 herbicides (glyphosate, metsulfuron‐methyl, ioxynil + bromoxynil, clopyralid) at vegetative and reproductive growth stages and found that, generally, the sensitivity of vegetative endpoints (e.g., biomass) and reproductive endpoints (e.g., number and germinability of seeds) was equivalent or vegetative endpoints were more sensitive among the herbicide and plant species combinations tested. Annual species were found to be more sensitive than perennial species, and in most cases, effects at the juvenile stage were found to be more sensitive than those observed at the reproductive phase (Mathiassen et al. 2021). Additionally, Christl et al. (2020) conducted a review comparing the sensitivity of vegetative and reproductive endpoints for both juvenile and mature plants and found that reproductive endpoints were similarly sensitive relative to vegetative endpoints derived in standard NTTP studies within a factor of 1.5. The authors also noted that there were no outliers among family, genus, or species in the analysis or among herbicidal modes of action, concluding that vegetative endpoints are protective of reproductive endpoints in the existing risk paradigm (Christl et al. 2020). It is important to point out that standard vegetative vigor and seedling emergence NTTP studies used to support herbicide/pesticide registration also consider shoot length and visual symptoms of injury in addition to biomass. These standard test species are also selected to be broadly representative of NTTPs in general, including annuals and perennials, monocots, and dicots, and reflective of a range in sensitivity depending on the mode of action. In a comparison of intrinsic sensitivity between standard NTTP crop test species and wild species using a variety of methods and endpoints, there was found to be no consistent differences, with crops species being slightly more sensitive by a factor of 1.4 based on multivariate regression analysis (Christl et al. 2019).

**Fig. 3:**
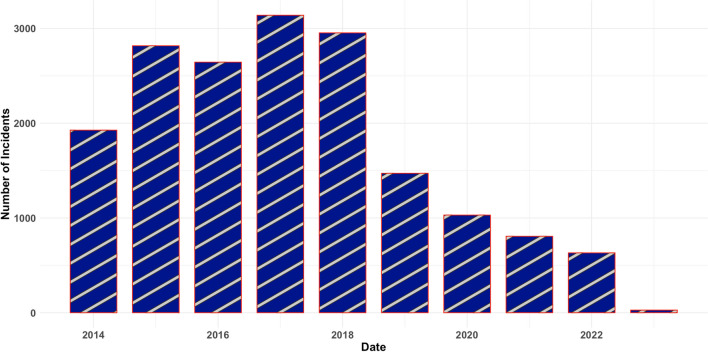
Incident reports logged in the USEPA’s Incident Data System (USEPA 2023b) indicating the date and number of individuals affected among a total of 2080 incidents classified as minor plant damage (IDS code PB) between 2013 and 2023

Although the Agency states that it has “limited confidence in the accuracy and validity” of the source data and recommends caution when analyzing the Incident Data System (USEPA 2023b), querying this information can be insightful with respect to the number and temporality of incidents reported, the alleged chemical responsible, and the number of individuals reportedly affected. For the past 10 years (2013–2023), the IDS logged a total of 2080 incidents classified as minor plant damage (IDS code PB; see Supplemental Information for more detail). Among these, 1.4% of cases reported effects to 100 individuals or more, whereas nearly 90% of incidents reported effects to 10 individuals or less, and 55% of reported incidents identified effects to just a single individual. Metsulfuron was alleged to be associated with incidents reporting the 1st and 3rd greatest number of individuals affected (1705 and 1067, respectively), and dicamba was allegedly associated with the 2nd most (1390). Zeta-Cypermethrin was alleged as the causal agent for the 4th greatest number of individuals affected (456), though this seems erroneous given the mode of action (insecticide affecting neuronal channels; γ-aminobutyric acid [GABA]). The balance of the top 10 is comprised primarily of auxins (2,4-D, Mecoprop-P, and MCPA). This information is summarized in the Supplemental Information and presented graphically in Fig. 3. Incidents reporting the most significant number of individuals affected took place in 2015 (April), 2017 (July), and 2016 (July), though there are no other obvious temporal trends with respect to number of incidents and individuals affected. Albeit an imperfect system that may be anecdotal in nature, given that there are nearly 400 million acres of cropland in the U.S. at present, most of which is conventional (vs. organic) agriculture, receiving at least some form of pesticide application annually, the number of incidents reported suggests that there are not widespread or frequent observations of NTTP effects. Moreover, just for the sake of argument, 2080 incidents averaged over 10 years would be ~ 200 incidents per year, and relative to the number of farms currently in operation (~ 2 million), that would be crudely equivalent to an incident frequency of 0.01%.

## Conclusions

Habitat alteration and non-native species are the most important drivers of the decline in native terrestrial plant diversity in North America according to the recovery plans assembled by the regulatory authorities in the U.S. and Canada. It is also important to note that no listed species in the U.S. or Canada faced a singular threat. In the recovery plans for all listed species, several threats were identified as contributing to their risk of extirpation or extinction. The recovery plans also indicate that pesticides, specifically herbicides, represent a micro-scale contributor to the decline of plant biodiversity in North America relative to other drivers. Despite the fact that habitat loss, non-native (particularly invasive) species, and climate change pose significantly greater threats to native plants relative to pesticides, or agriculture in general at this point, there is a disproportionate public fixation on the latter. We, as a society, appear to be at risk of missing the forest for the trees. Clearly, pesticides have a contentious origin (Brain and Anderson 2020), plausible biological relevance (BCPC 2018; Carson 1962; Fukuto 1990), and an easily exploited legal construct in the United States. (e.g., citizen suit provisions (see Clean Water Act (CWA): USEPA 2002; Endangered Species Act (ESA): USFWS 1973) making them an easy target for litigious opportunism. However, being an easy target does not mean pesticides are the right target. Granted, it is difficult to sue the Spanish conquistador Hernando de Soto for having introduced feral hogs to Florida in 1539 for example, or John Rolfe for introducing a new variety of tobacco in1612, or Mother Nature for pathogen outbreaks, droughts, and floods. The unintended consequences of promoting a narrative and sensationalizing a micro-scale contributor to a macro-scale issue include lack of public awareness regarding the primary drivers of species decline, missed opportunity to proactively address them, and potential misallocation of resources. In effect, metaphorically, we will miss the conservation opportunity of the forest for the counterfactual narrative of the trees.

### Supplementary Information

Below is the link to the electronic supplementary material.

Supplementary file1 (DOCX 5439 KB)

## Data Availability

The data generated from this study is available in the supplementary information.

## Declarations

This study did not involve humans or animals.
