# Supplementary material for: Where have all the flowers gone? A systematic evaluation of factors driving native terrestrial plant decline in North America
Source: Environ Sci Pollut Res Int. 2024 Jul 20;31(35):48460–83. doi: 10.1007/s11356-024-34349-9 (PMC11297832; doi:10.1007/s11356-024-34349-9)
Supplement: Supplementary file 1 — Supplementary file1 (DOCX 5439 KB) [file 11356_2024_34349_MOESM1_ESM.docx]

**Supplementary Information**

**Where Have All the Flowers Gone? A Systematic Evaluation of Factors Driving Native Terrestrial Plant Decline in North America**

Ryan S. Prosser^1^ and Richard A. Brain^2^

^1^University of Guelph, School of Environmental Sciences, Guelph, ON, Canada

^2^Syngenta Crop Protection LLC, Greensboro, NC, USA

**Table S1.** The number of threatened or endangered terrestrial plant species in the **United States** affected by different drivers of decline according to the United States Fish and Wildlife Service. The drivers of decline for each listed plant species were listed in importance from primary to quinary.

|  | **Primary** | **Secondary** | **Tertiary** | **Quaternary** | **Quinary** | **Total Number of Species** |
| --- | --- | --- | --- | --- | --- | --- |
| **Habitat alteration** | 240 | 141 | 63 | 3 | 0 | 447 |
| **Development** | 217 | 142 | 60 | 10 | 0 | 429 |
| **Non-native species** | 411 | 141 | 94 | 37 | 16 | 699 |
| **Military activity** | 1 | 12 | 53 | 25 | 2 | 93 |
| **Climate change** | 6 | 23 | 66 | 66 | 30 | 191 |
| **Disease** | 3 | 41 | 10 | 12 | 17 | 83 |
| **Extreme weather** | 2 | 59 | 60 | 27 | 12 | 160 |
| **Drought** | 4 | 10 | 14 | 5 | 1 | 34 |
| **Harvesting** | 18 | 36 | 16 | 15 | 3 | 88 |
| **Recreational activities** | 14 | 55 | 61 | 45 | 23 | 198 |
| **Fire** | 13 | 144 | 86 | 43 | 5 | 291 |
| **Herbicides** | 2 | 9 | 5 | 5 | 3 | 24 |
| **Pesticides** | 0 | 0 | 7 | 7 | 4 | 18 |
| **Insecticides** | 0 | 1 | 1 | 2 | 0 | 4 |

**Table S2.** The number of endangered, threatened, or species concern terrestrial plant species in **Canada** affected by different drivers of decline according to the Government of Canada. The drivers of decline for each listed plant species were listed in importance from primary to quinary.

|  | **Primary** | **Secondary** | **Tertiary** | **Quaternary** | **Quinary** | **Total Number of Species** |
| --- | --- | --- | --- | --- | --- | --- |
| **Habitat Alteration** | 70 | 28 | 8 | 2 | 1 | 109 |
| **Development** | 46 | 37 | 9 | 4 | 4 | 100 |
| **Non-native Species** | 40 | 40 | 22 | 9 | 3 | 114 |
| **Military Activity** | 0 | 0 | 0 | 0 | 0 | 0 |
| **Climate Change** | 10 | 8 | 9 | 9 | 3 | 39 |
| **Disease** | 4 | 2 | 2 | 1 | 0 | 9 |
| **Extreme Weather** | 3 | 2 | 2 | 1 | 0 | 8 |
| **Drought** | 0 | 0 | 0 | 1 | 0 | 1 |
| **Harvesting** | 3 | 5 | 4 | 1 | 0 | 13 |
| **Recreational activities** | 11 | 27 | 25 | 11 | 0 | 74 |
| **Fire** | 6 | 11 | 17 | 6 | 2 | 42 |
| **Herbicides** | 0 | 2 | 4 | 4 | 0 | 10 |
| **Pesticides** | 0 | 0 | 0 | 0 | 0 | 0 |
| **Insecticides** | 0 | 0 | 0 | 0 | 0 | 0 |

**Table S3.** The number of threatened or endangered terrestrial plant species in the United States according to United States Fish and Wildlife region.

| **Region** | **Geographic Region** | **Number of Species** |
| --- | --- | --- |
| 1 | Pacific | 458 |
| 2 | Southwest | 65 |
| 3 | Midwest | 16 |
| 4 | Southeast | 168 |
| 5 | Northeast | 15 |
| 6 | Mountain-Prairie | 35 |
| 7 | Alaska | 1 |
| 8 | Pacific Southwest | 181 |

**Table S4.** The threatened or endangered terrestrial plant species in the **United States** for which drivers of decline could not be identified.

| **Scientific name** | **Common name** | **Listing Status** |
| --- | --- | --- |
| *Chamaesyce deltoidea serpyllum* | Wedge spurge | Endangered |
| *Chromolaena frustrata* | Cape Sable Thoroughwort | Endangered |
| *Delphinium variegatum ssp. kinkiense* | San Clemente Island larkspur | Endangered |
| *Harrisia (=Cereus) aboriginum (=gracilis)* | Aboriginal Prickly-apple | Endangered |
| *Linum arenicola* | Sand flax | Endangered |
| *Malacothamnus clementinus* | San Clemente Island bush-mallow | Endangered |
| *Sidalcea nelsoniana* | Nelson's checker-mallow | Threatened |

**Table S5.** The endangered, threatened, or species concern terrestrial plant species in the **Canada** for which drivers of decline could not be identified.

| **Scientific name** | **Common name** | **Listing Status** |
| --- | --- | --- |
| *Salix jejuna* | Barrens Willow | Endangered |
| *Phegopteris hexagonoptera* | Broad Beech Fern | Special Concern |
| *Isoetes engelmannii* | Engelmann's Quillwort | Endangered |
| *Astragalus robbinsii var. fernaldii* | Fernald's Milk-vetch | Special Concern |
| *Agalinis gattingeri* | Gattinger's Agalinis | Endangered |
| *Arisaema dracontium* | Green Dragon | Special Concern |
| *Eleocharis equisetoides* | Horsetail Spike-rush | Endangered |
| *Carex juniperorum* | Juniper Sedge | Endangered |
| *Erigeron philadelphicus var. provancheri* | Provancher's Fleabane | Special Concern |
| *Sanicula bipinnatifida* | Purple Sanicle | Threatened |
| *Ammannia robusta* | Scarlet Ammannia | Endangered |
| *Quercus shumardii* | Shumard Oak | Special Concern |

**Figure S1.** This map provides a visual representation of the varying terrestrial and freshwater ecosystems in Canada. The map delineates ecoprovinces with a thin black boundary line and ecoprovinces are labeled with a code. Fine black dashed lines show provincial and territorial boundaries. Below the map, ecoprovince codes and their corresponding names are listed for reference. Ecosystem data here represent the latest comprehensive and comparable estimates for the country for the time period the data were developed. Data for built-up and artificial surfaces are from 2010.


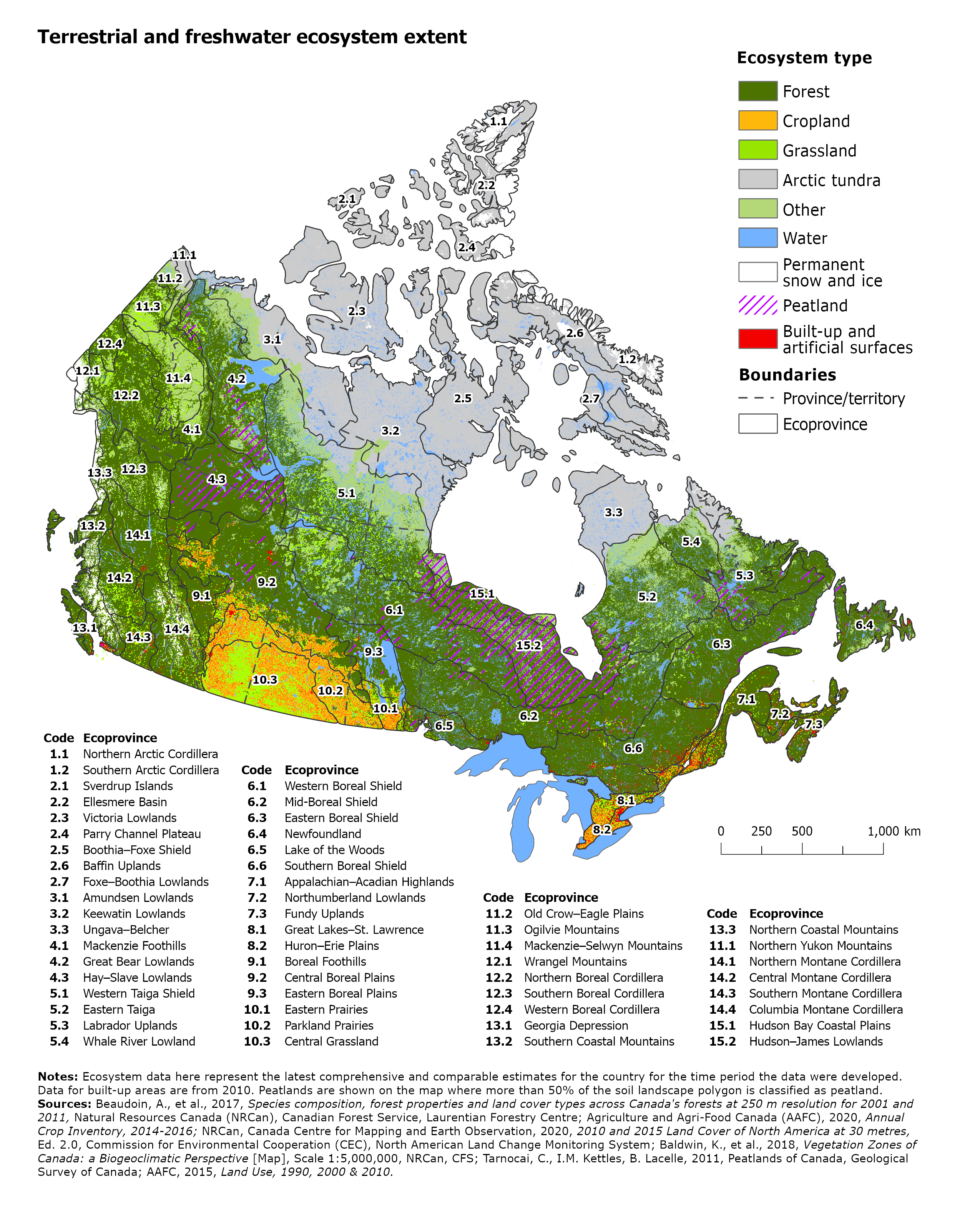


Sources:

Beaudoin, A., et al., 2017, Species composition, forest properties and land cover types across Canada's forests at 250 m resolution for 2001 and 2011, Natural Resources Canada (NRCan), Canadian Forest Service, Laurentian Forestry Centre, https://doi.org/10.23687/ec9e2659-1c29-4ddb-87a2-6aced147a990 (accessed March 31, 2019);

Agriculture and Agri-Food Canada (AAFC), 2020, Annual Crop Inventory, 2014-2016, https://open.canada.ca/data/en/dataset/ba2645d5-4458-414d-b196-6303ac06c1c9 (accessed December 3, 2020);

NRCan, Canada Centre for Mapping and Earth Observation, 2020, 2010 and 2015 Land Cover of North America at 30 metres, Ed. 2.0, Commission for Environmental Cooperation (CEC), North American Land Change Monitoring System, http://www.cec.org/north-american-land-change-monitoring-system/ (accessed December 9, 2020);

Baldwin, K., et al., 2018, Vegetation Zones of Canada: a Biogeoclimatic Perspective [Map], Scale 1:5,000,000, NRCan, CFS, https://open.canada.ca/data/en/dataset/22b0166b-9db3-46b7-9baf-6584a3acc7b1 (accessed October 26, 2020);

Tarnocai, C., I.M. Kettles, B. Lacelle, 2011, Peatlands of Canada, Geological Survey of Canada, https://geoscan.nrcan.gc.ca/starweb/geoscan/servlet.starweb?path=geoscan/fulle.web&search1=R=288786 (accessed March 16, 2020);

AAFC, 2015, Land Use, 1990, 2000 & 2010, https://open.canada.ca/data/en/dataset/18e3ef1a-497c-40c6-8326-aac1a34a0dec (accessed June 1, 2020).
